# An integrated approach for prescribing fewer chest x-rays in the ICU

**DOI:** 10.1186/2110-5820-1-4

**Published:** 2011-03-21

**Authors:** Vincent Ioos, Arnaud Galbois, Ludivine Chalumeau-Lemoine, Bertrand Guidet, Eric Maury, Gilles Hejblum

**Affiliations:** 1 Hôpital Delafontaine, Service de Réanimation Polyvalente, Saint-Denis F-93205, France; 2 AP-HP, Hôpital Saint-Antoine, Service de Réanimation Médicale, Paris F-75012, France; 3 UPMC Univ Paris 06, UMR_S 938, CdR Saint-Antoine, F-75005, Paris, France; 4 INSERM, UMR_S 938, CdR Saint-Antoine, F-75012, Paris, France; 5 Institut Gustave Roussy, Service de Réanimation Médico-Chirurgicale, F-94805, Villejuif, France; 6 UPMC Univ Paris 06, UMR_S 707, Paris F-75012, France; 7 INSERM, U707, Paris F-75012, France; 8 AP-HP, Hôpital Saint-Antoine, Unité de Santé Publique, Paris F-75012, France

## Abstract

Chest x-rays (CXRs) are the main imaging tool in intensive care units (ICUs). CXRs also are associated with concerns inherent to their use, considering both healthcare organization and patient perspectives. In recent years, several studies have focussed on the feasibility of lowering the number of bedside CXRs performed in the ICU. Such a decrease may result from two independent and complementary processes: a raw reduction of CXRs due to the elimination of unnecessary investigations, and replacement of the CXR by an alternative technique. The goal of this review is to outline emblematic examples corresponding to these two processes. The first part of the review concerns the accumulation of evidence-based data for abandoning daily routine CXRs in mechanically ventilated patients and adopting an on-demand prescription strategy. The second part of the review addresses the use of alternative techniques to CXRs. This part begins with the presentation of ultrasonography or capnography combined with epigastric auscultation for ensuring the correct position of enteral feeding tubes. Ultrasonography is then also presented as an alternative to CXR for diagnosing and monitoring pneumothoraces, as well as a valuable post-procedural technique after central venous catheter insertion. The combination of the emblematic examples presented in this review supports an integrated global approach for decreasing the number of CXRs ordered in the ICU.

## Introduction

Among investigations performed daily in the Intensive Care Unit (ICU), bedside chest x-rays (CXRs) are completely trivialized. However, such CXRs are sources of discomfort and irradiation for the patients, of disorganization of the radiology department, and of potential risk of accidental removal of devices (catheters, tubes) and microbial dissemination, all resulting in additional cost for the community. In this context, it is essential to assess whether it is possible to reduce the number of CXRs performed during an ICU stay without impairing the quality of care.

There is a great variability of prescription practices from one team to another, because the individual perception of practitioners about what is appropriate is based on personal experience or expert recommendations. Indications for ordering CXRs in ICUs have been poorly studied in a systematic way. Apart from invasive procedures that are easier to study [1-3], research has mainly focussed on prescribing strategies (i.e., routine vs. on-demand) [4-12] more than on precise clinical contexts.

A study that collected the opinions of 82 ICU physicians on CXR indications [8] illustrates the above-mentioned variable perceptions. The study proposed a questionnaire composed of 29 items relative to the placement of medical devices and their surveillance, as well as various clinical situations. The study was based on the Delphi method (anonymous and iterative collection of the answers with feedback of the collected answers at each iteration) and was designed to estimate the consensus on indications of CXR prescription in various clinical situations. Physicians' opinions about the appropriateness of a systematic prescription of CXRs in the proposed situations were collected through a 1 to 9 scoring scale during iterative sequences of interrogation using a dedicated Web application. A strong consensus was observed--i.e., low variability of the answers together with a low or high median score--for 10 questions that represented widely accepted reasonable attitudes. The study evidenced the importance of the clinical context in the decision of prescription and the difficulty in making too general recommendations not taking into account the heterogeneity of the clinical scenarios.

The present article is not a systematic review but was designed to outline the two complementary processes that should be considered for decreasing CXR ordering. On the one hand, fewer CXRs may result from the raw elimination of some investigations performed in patients, the objective being to merely reduce the rate of unnecessary investigations. Because most articles on this topic concern the current debate of whether mechanically ventilated patients should receive routine daily CXRs or on-demand CXRs, we will focus on this particular question. On the other hand, fewer CXRs may result from utilization of alternative techniques in specific indications. We present and discuss emblematic situations for which such alternative techniques have been proposed. In that regard, CT scans cannot be viewed as routine investigations and therefore will not be considered in this presentation as an alternative to CXRs.

## Reduction of the number of unnecessary CXRs ordered in patients on mechanical ventilation

The American College of Radiology recommends routine daily chest radiographs for mechanically ventilated patients, and use of additional CXRs if necessary [13]. This strategy is controversial [5,8,11,12,14,15]; some authors support it [7,16,17], whereas others advocate prescription of chest radiographs only when warranted by the patient's clinical status [5,8,9,11,12,18]. The above-mentioned Delphi study revealed that physicians' opinions on the appropriateness of routine CXRs in all patients on mechanical ventilation considerably vary from a physician to another [8].

Routine CXRs theoretically have two main advantages. First, some potentially life-threatening situations that might otherwise be missed could be discovered and treated. Second, scheduling CXRs during morning rounds might be more efficient on a logistical point of view. In contrast, the on-demand strategy might avoid unnecessary radiation exposure and provides substantial cost savings [19], but an increased number of CXRs might be needed during the rest of the day to compensate for those not done in the morning.

A recent meta-analysis selected eight studies that compared on-demand and daily routine strategies, including a total of 7,078 patients [20]. No difference in ICU mortality, ICU length of stay, and duration of mechanical ventilation was found between the on-demand and daily routine groups, and the meta-analysis highly suggests abandoning routine CXRs. However, only two small-sized (n = 165 and n = 94) and single-center, randomized, controlled trials [5,11] were included in this meta-analysis. As a consequence, this meta-analysis lacks powerful enough evidence for totally convincing ICU physicians to abandon daily routine CXRs [21].

**Figure 1:**
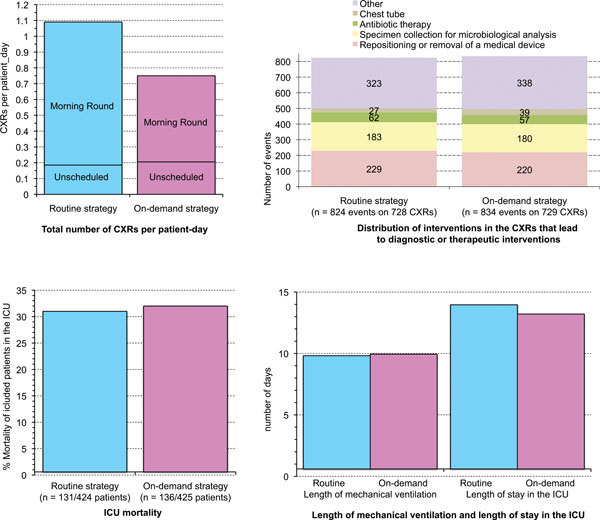
**Main results of the RARE study **[22].

Nevertheless, while this meta-analysis was in the process of being published, the RARE study [22], based on a cluster-randomized crossover design and involving 849 patients and 7,755 CXRs, compared routine and on-demand prescription strategies in ICU patients on mechanical ventilation. With the "routine strategy", CXRs were performed daily in patients on mechanical ventilation, irrespective of their clinical status, during a morning round CXR session. With the "on-demand strategy", CXRs were performed in this morning round session if warranted by the clinical examination and the analysis of biological parameters. Twenty-one ICUs (medical, surgical or medico-surgical) in 18 hospitals (teaching and nonteaching) were randomly assigned to use "routine" or "on-demand" strategy during the first of two treatment periods. All the ICUs used the alternative strategy in the second period. The primary outcome measure was the mean number of CXRs per patient-day of mechanical ventilation. Secondary outcome measures were related to the quality and safety of care (days of mechanical ventilation, ICU length of stay, and ICU mortality). Moreover, the number of unscheduled CXRs performed was analyzed, as well as the diagnostic and therapeutic impact of the CXRs performed within each strategy. The results of the study are summarized in Figure 1. During the study period, 424 patients had 4,607 routine CXRs (mean per patient-day of mechanical ventilation 1.09; 95% confidence interval (CI, 1.05-1.14), and 425 had 3,148 on-demand CXRs (mean 0.75; 95% CI, 0.67-0.83), which corresponded to a reduction of 32% (95% CI, 25-38) with the on-demand strategy (*p *< 0.0001). Duration of mechanical ventilation as well as ICU length of stay and ICU mortality did not significantly differ between the two groups. The difference in the total number of routine and on-demand CXRs was not significant when the analysis was restricted to CXRs with new findings that led or contributed to diagnostic procedures or therapeutic interventions.

Finally, there was no increase in the number of unscheduled CXRs performed in the afternoon or in the night in the on-demand strategy, and therefore no disruption in the organisation of the medical imaging department. This study strongly suggests that routine daily CXRs in the ICU patient on mechanical ventilation should be abandoned. The support for the on-demand restrictive strategy is in line with previous studies that had some methodology flaws [20]. The main limit to its broad application lies in the fact that French ICUs are closed units and the results may not be applicable to open ICUs, an organization model found in other countries [23]. In that regard, it is worth mentioning that the Haute Autorité de Santé (French Health Authority) currently does not recommend a daily routine CXR in all mechanically ventilated patients but only in particular cases of such patients [24].

## Alternatives to CXR when an imaging control is needed

Some situations in ICU require an imaging control usually relying on a CXR. In France, the Haute Autorité de Santé indicates that, for instance, a control after placement of a thoracic drain or patient's intubation is an indication for a CXR [24]. However, in situations further detailed, alternative techniques involving fewer disadvantages than CXR have been recently proposed. Some intensivists might be reluctant to avoid CXRs in these situations because it might be a piece of evidence in case of litigation. However, if the findings issued from these well-assessed alternative techniques are appropriately documented in the patient's chart, such a fear should not be a bridle to their utilization. Moreover, if the alternative technique is ultrasonography, recording or printing images is a basic functionality available in most ultrasound scanners.

### Alternatives to CXR for ensuring correct placement of enteral feeding tube

The collected opinions of ICU physicians on the appropriateness of a systematic CXR after placement of a nasogastric tube for enteral nutrition were highly variable [8]. However, ensuring correct enteral feeding tube (EFT) position is of paramount importance for patients in the ICU. Accidental placement of EFT in the tracheobronchial tract can lead to potentially lethal complications and tracheal intubation does not always prevent this misplacement [25]. When used alone, epigastric auscultation after air injection through the EFT is not a reliable test for confirming the adequate placement of EFT [26-28]. Some studies have suggested testing the pH of an aspirate obtained from the EFT to ensure proper placement, but this test can be inconclusive in patients with small-bore EFT or those on acid suppression therapies [26]. Therefore, most guidelines recommend confirmation of EFT placement with a CXR before starting enteral nutrition [28,29]. Nevertheless, two interesting alternatives to CXR might be considered: ultrasonography and capnography combined with epigastric auscultation.

**Figure 2:**
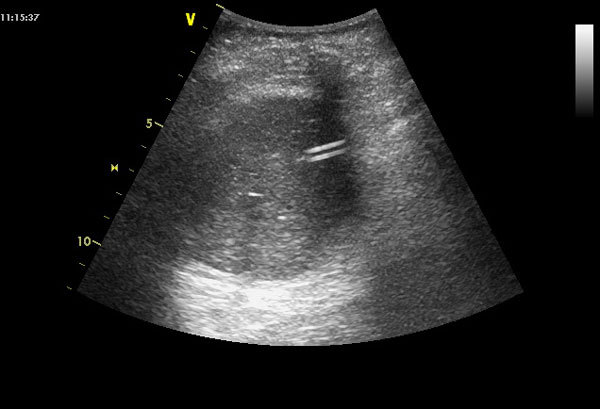
**Assessment of intragastric position of a small bore enteral feeding tube by ultrasonography **[31]. The probe is placed in the middle epigastric area and oriented toward the left upper abdominal quadrant to visualize the gastric area. The small bore feeding tube appears as two parallel hyperechogenic lines.

Bedside ultrasonography is a noninvasive procedure increasingly used in ICU by nonradiologist physicians who can obtain reliable results after a short training in various organs exploration [30,31]. Within 5 minutes, a 2- to 5-MHz probe-based ultrasonography was shown to allow the display of a small-bore EFT in the digestive tract with a sensitivity of 97% and to assess whether it is properly placed in the stomach (Figure 2) [32]. If the EFT is not immediately visible by ultrasound, injection of 5 ml of normal saline mixed with 5 ml of air into the tube increases the sensitivity. This radiation-free procedure is more rapid than conventional radiography and can be taught to ICU physicians during a short training period [32]. Radiography might be only reserved for the rare cases of ultrasonography failures, due to gas interposition, for example.

Capnography often is used to assess expiratory CO_2_. However, it is possible to connect the capnography device to the EFT via the tip of an endotracheal tube and to assess the correct placement of the EFT by the absence of CO_2 _detection. The EFT must be inserted to a depth of 30 cm from the nostril and should not get coiled in the pharynx. When the EFT is accidentally inserted into the respiratory tract, the capnograph displays a normal capnogram, whereas when the EFT is inserted into the esophagus, the capnograph does not display a CO_2 _waveform [33]. EFT permeability is essential for CO_2 _detection. In our ICU, we ensure this permeability by removing the guidewire, insufflating and then exsufflating air with a 50-ml syringe, before connecting the capnography device. We use a colorimetric capnography device after a 30-cm insertion and then we complete the insertion until 50 cm from the nostril. Finally, to check that the EFT is not coiled in the esophagus after its complete insertion, nurses perform epigastric auscultation. Radiography is required only when epigastric auscultation is inconclusive (10.1% of cases). This local protocol combining colorimetric capnography and epigastric auscultation had a perfect specificity to confirm correct EFT placement, improves nurse's organization of care, saves time, and decreases costs [34,35]. Another advantage of this procedure is that the accidental tracheobronchial insertion is detected after 30-cm insertion. Therefore, the procedure also prevents all risks of pneumothorax or hydrothorax--rare but potentially fatal complications of EFT misplacement not prevented by a postprocedural radiography.

### Alternative to CXR to diagnose and monitor pneumothorax

Many pneumothoraces (30% to 72%) are not seen by CXRs because of their anterior location [36]. This phenomenon of radio-occult pneumothoraces is not explained by too small to been seen pneumothoraces because 50% of occult pneumothoraces can be with tension [37]. Pleural ultrasonography has greater sensitivity than CXR for pneumothorax diagnosis in patients in ICUs or in trauma centres and after pleural biopsy [36,38-41]. In the retrospective study by Lichtenstein and colleagues, ultrasonography detected all pneumothoraces in ICU patients, including those not identified by CXR [38]. Ultrasound diagnosis of pneumothorax relies on three signs: abolition of lung sliding, the A-line sign, and the lung point.

**Figure 3:**
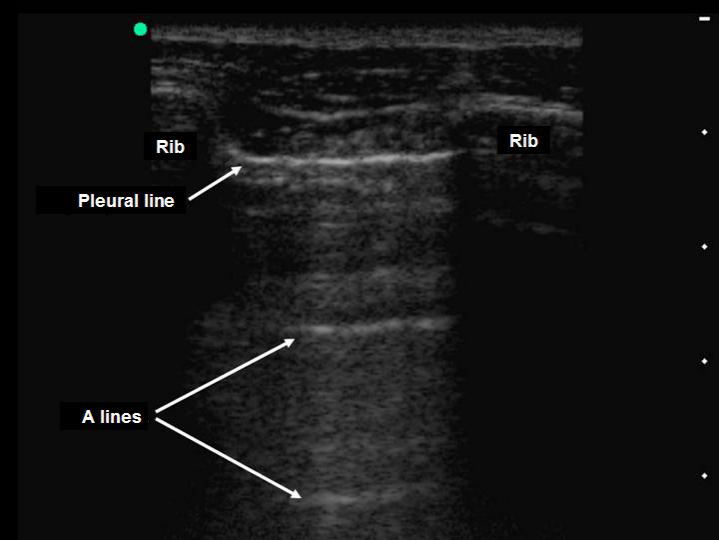
**Pleural ultrasonography in two-dimensional mode **[31]. The pleural line is seen between two ribs. Lung sliding is abolished when both the parietal and visceral pleura do not slide while the patient is breathing. The A-line sign corresponds to the presence of linear horizontal artefacts at regular intervals below the pleural line (A-lines) without B-lines. The A-line sign is part of the ultrasound semiology of the normal lung and pneumothorax. Reproduced with permission (ACCP - Chest).

**Figure 4:**
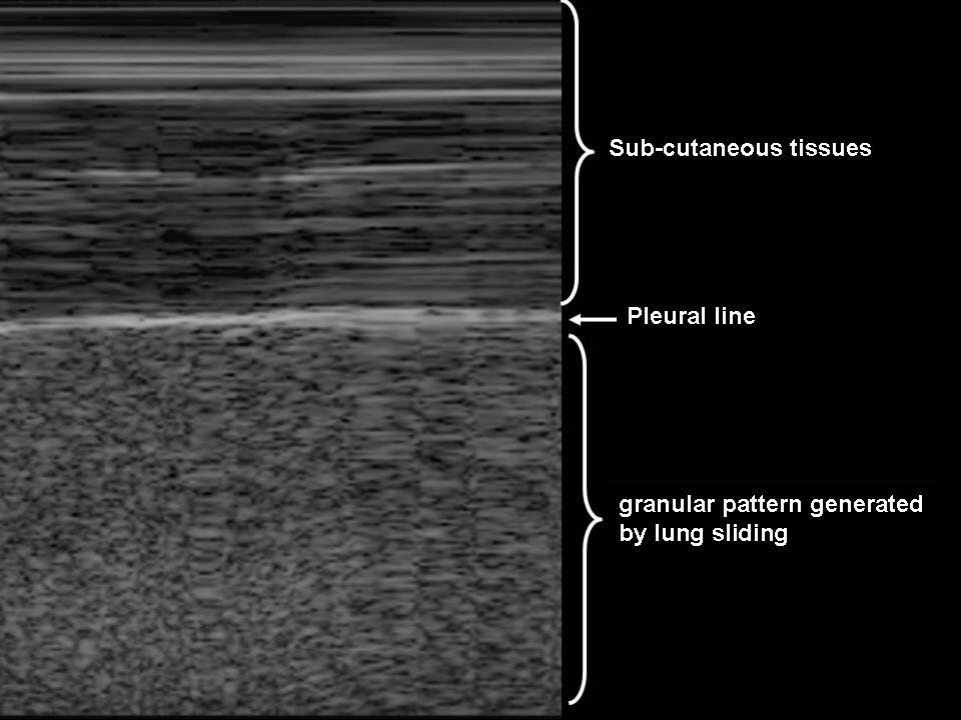
**Assessment of lung sliding on pleural ultrasonography in time-motion mode on a patient without pneumothorax **[31]. Lung sliding generates a granular pattern under the pleural line. Subcutaneous tissue over the pleural line does not move while the patient is breathing, generating horizontal lines. Reproduced with permission (ACCP - Chest).

**Figure 5:**
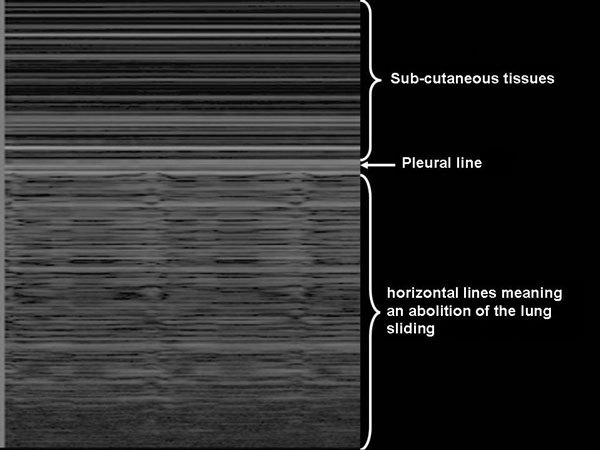
**Abolition of lung sliding on pleural ultrasonography in time-motion mode in a patient with pneumothorax **[31]. While the patient is breathing, the (normal) granular pattern under the pleural line is replaced by horizontal lines, indicating abolition of lung sliding. Reproduced with permission (ACCP - Chest).

The abolition of lung sliding has a perfect sensitivity (100%) for the diagnosis of pneumothorax, but its specificity ranges from 78% to 91% when controls are ICU patients or have normal lungs, respectively (Figures 3, 4, 5) [42,43]. Actually, the abolition of the lung sliding can be present in many other situations than pneumothorax (e.g., acute respiratory distress syndrome, atelectasia, apnea, pleurodesis) [44]. Thus, the presence of a lung sliding allows ruling out a pneumothorax, whereas the abolition of the lung sliding cannot affirm it.

**Figure 6:**
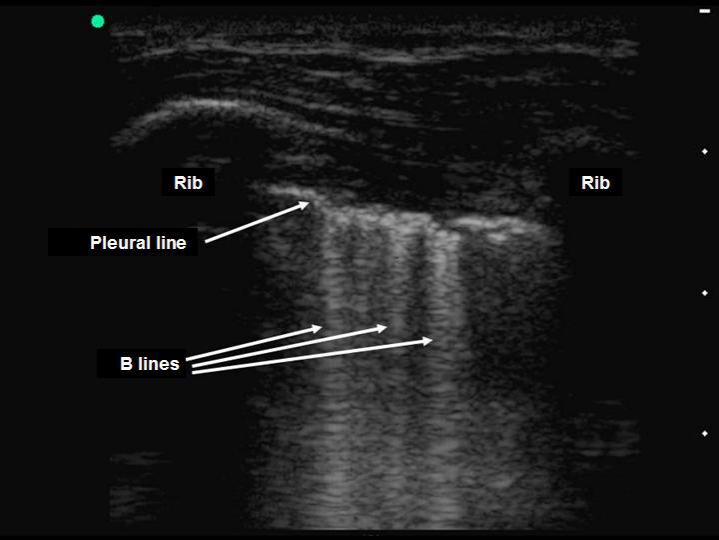
**Detection of B-lines on pleural ultrasonography in two-dimensional mode**. The presence of vertical linear artefacts arising from the pleural line (B-lines or comet-tail artefacts) rules out pneumothorax in this patient with interstitial syndrome. Reproduced with permission (ACCP - Chest).

The presence of horizontal linear artefacts at regular intervals below the pleural line (A-lines) is part of the ultrasound semiology of normal lungs and pneumothorax (Figure 3). In contrast, vertical linear artefacts arising from the pleural line, i.e., B-lines, are observed when alveolar-interstitial syndrome is present, as well as in the last two intercostal spaces in 27% of healthy subjects (Figure 6) [45]. The A-line sign is defined as the presence of A-lines without B-lines (Figure 3) and has a sensitivity of 100% and a specificity of 60% for the diagnosis of pneumothorax. The presence of B-lines rules out pneumothorax diagnosis, whereas the absence of B-lines cannot affirm it [46].

The lung point is detected while the probe is stationary: there is lung sliding during inspiration (when the lung contacts the wall), which disappears during expiration (when the lung is not in contact with the wall). Its sensitivity for diagnosis of pneumothorax is 66% and its specificity is 100% [43]. The lung point is an inconstant sign but constitutes the only ultrasonographic sign able to affirm the presence of a pneumothorax.

In the Delphi study mentioned earlier, most ICU physicians supported a daily routine CXR in patients with a chest tube [8]. However, after drainage, ultrasonography is better than CXR for detecting residual pneumothoraces, whereas 39% of them are not identified by CXR [31]. After drainage of primary spontaneous pneumothoraces, performance of ultrasonography is excellent [31]. After drainage of nonprimary spontaneous pneumothorax, the positive predictive value of ultrasonography was 100% in the presence of a lung point. However, it decreased to 90% in the absence of a lung point [31]. Exclusive use of ultrasonography for follow-up of nonprimary spontaneous pneumothorax seems possible, but the physician must be aware that in the absence of lung point, diagnosis of pneumothorax should not be made if other causes of lung sliding abolition have not been ruled out. We recommend performing a CT scan if doubt persists, especially if new chest tube insertion is under consideration.

These excellent performances make pleural ultrasonography more than an alternative to CXR and should be considered as the "bedside gold standard" to diagnose and monitor pneumothorax. Moreover, ultrasonography gives faster results than CXR and is performed competently by naïve physicians after a brief training session [31,47].

### Alternative to CXR after central venous catheter insertion

The French ICU physicians who participated in the Delphi study agreed on the appropriateness of performing a CXR after central venous catheter (CVC) insertion in the superior vena cava system [8]. After catheterization of the subclavian or internal jugular vein, CVC tip misplacement occurs in 5% to 6% and pneumothorax occurs in 1.5% to 3.1% and 0.1% to 0.2%, respectively [48].

Clinical evaluation of the patient to predict the absence of complications after CVC insertions via the subclavian vein or internal jugular vein was very accurate in Gray and colleagues' study [49]. However, Gladwin and colleagues showed that the clinical impression of the operator (based on the number of needle passes, difficulty establishing access, operator experience, poor anatomical landmarks, number of previous catheter placements, resistance to wire or catheter advancement, resistance to aspiration of blood or flushing of the catheter ports, sensations in the ear, chest, or arm, and development of signs or symptoms suggestive of pneumothorax) had a poor sensitivity (44%) and specificity (55%) for predicting a complication [50]. Gladwin and colleagues concluded that postprocedural CXR remains necessary because clinical factors alone cannot reliably identify tip misplacement.

Nevertheless, as mentioned, numerous pneumothoraces can be missed by bedside CXR, whereas ultrasonography showed excellent sensitivity and specificity for diagnosing pneumothorax within a few minutes. Postprocedural ultrasonography and CXRs were compared after insertion of 85 central venous catheters (70 subclavian and 15 internal jugular) [51]. Ultrasonic examination feasibility was 99.6%. Ten misplacements and one pneumothorax occurred. This pneumothorax and all misplacements except one were diagnosed by ultrasound. Taking into consideration signs of misplacement and pneumothorax, ultrasonic examination did not give any false-positive results. Moreover, ultrasound guidance increases the success rate of CVC insertion, saves time, and decreases the complication rate [52]. Considering these results, it appears logical to use the same ultrasonographic device to assess both the adequate position of the CVC and the absence of pneumothorax after the procedure. The only limit of ultrasonography in this indication is the lack of visualization of azygos, internal thoracic and cardiophrenic veins, and an inconstant visualization of the superior vena cava. Thus, ultrasonography could be proposed for assessing the absence of misplacement and pneumothorax while limiting CXR requirement to incomplete ultrasonographic analysis.

## Conclusions

We have shown that bedside CXR could be avoided in many circumstances. This is true for most mechanically ventilated patients and for ensuring proper placement of devices, such as feeding tubes and central venous catheter. This restrictive policy for ordering bedside CXR requires an assessment of the patient's clinical status at least once a day before ordering CXR. It means that CXR should never replace clinical evaluation of the patient but should be prescribed on the basis of clinical suspicion. As a consequence, the organization of the ICU might have to be modified to allow the implementation of such a prescribing strategy and the reduction of the number of CXRs ordered. Ultrasonography is a very good alternative to CXR. For example, ultrasonography is more accurate than CXR for detecting pneumothorax. However, short training courses must be organized to reach a basic level of competency for every physician working in ICU. A policy of reducing the number of CXRs has many advantages (comfort for the patients, better organization of the radiology department, cost reduction) and should be widely implemented in the ICU. The emblematic examples presented in this review can be combined, and the global picture issued from this review suggests adopting an integrated approach for decreasing the number of CXR investigations performed in the ICU.

## Competing interests

The authors declare that they have no competing interests.

## Authors' contributions

VI, AG, LC-L, BG, EM and GH all participated in the design and in the redaction of the first draft of the article, corrected and approved the final version.
